# Origins of Superior Dynamic Visual Acuity in Baseball Players: Superior Eye Movements or Superior Image Processing

**DOI:** 10.1371/journal.pone.0031530

**Published:** 2012-02-22

**Authors:** Yusuke Uchida, Daisuke Kudoh, Akira Murakami, Masaaki Honda, Shigeru Kitazawa

**Affiliations:** 1 Faculty of Sport Sciences, Waseda University, Mikajima, Tokorozawa, Saitama, Japan; 2 Department of Ophthalmology, School of Medicine, Juntendo University, Hongo, Bunkyo-ku, Tokyo, Japan; 3 Department of Neurophysiology, School of Medicine, Juntendo University, Hongo, Bunkyo-ku, Tokyo, Japan; 4 Dynamic Brain Network Laboratory, Graduate School of Frontier Biosciences, Osaka University, Osaka, Japan; 5 Department of Brain Physiology, Graduate School of Medicine, Osaka University, Osaka, Japan; University of Alberta, Canada

## Abstract

Dynamic visual acuity (DVA) is defined as the ability to discriminate the fine parts of a moving object. DVA is generally better in athletes than in non-athletes, and the better DVA of athletes has been attributed to a better ability to track moving objects. In the present study, we hypothesized that the better DVA of athletes is partly derived from better perception of moving images on the retina through some kind of perceptual learning. To test this hypothesis, we quantitatively measured DVA in baseball players and non-athletes using moving Landolt rings in two conditions. In the first experiment, the participants were allowed to move their eyes (free-eye-movement conditions), whereas in the second they were required to fixate on a fixation target (fixation conditions). The athletes displayed significantly better DVA than the non-athletes in the free-eye-movement conditions. However, there was no significant difference between the groups in the fixation conditions. These results suggest that the better DVA of athletes is primarily due to an improved ability to track moving targets with their eyes, rather than to improved perception of moving images on the retina.

## Introduction

Dynamic visual acuity (DVA) is generally defined as the ability to discriminate the fine parts of a moving object during relative motion between the object and the observer [1], [2]. Previous studies reported that dynamic visual acuity correlates with performance in ball games such as volleyball and basketball [3], baseball [4], softball [5], motorsports [6], and catching tasks [7]. In addition, baseball, tennis, and badminton players generally display superior DVA to non-athletes in that they are able to recognize the gap in the Landolt “C” ring at significantly higher velocities than non-athletes [4], [8].

Some studies have suggested that the superior DVA of athletes reflects their superior ability to track moving objects by making appropriate saccadic eye movements [9]–[11]. For example, Land and McLeod [10] showed that a professional cricket player made exact anticipatory saccades to the bounce point, whereas an amateur player waited until the ball completed a large part of its flight to the bounce point before starting the saccade. Major league baseball players also make anticipatory saccades to balls travelling faster than the upper limit of eye movements as the ball approaches the batter's box [9], [12].

On the other hand, it is well known that visual perception improves as a function of past experience [13], [14]. This process, which is termed perceptual learning, takes place during motion perception as well when participants are exposed to motion stimuli without moving their eyes [15]–[19]. As athletes who play ball games are repeatedly exposed to motion stimuli during their training, repeated exposure to motion stimuli is likely to improve their perception of moving objects independently of improvements in eye movements. We thus suggest another possible reason for the superior DVA of athletes. In ordinary participants, retinal image motion of just a few degrees per second measurably reduces visual acuity [20], [21], but it is possible that the range of acceptable motion might be larger in athletes as a result of perceptual learning of visual perception.

To test this hypothesis, we decided to measure the DVA of skilled baseball players and age-matched control participants while they viewed a moving Landolt ring in two conditions, free-eye movement conditions and fixation conditions. If the baseball players displayed better DVA in both conditions, this would suggest that skilled players are more able to tolerate image motion on their retinas. However, if they only displayed better DVA in the free-eye movement conditions then their superior DVA could be solely attributed to better eye movement control.

## Methods

### 2-1. Participants

Sixteen males participated in this study. Eight belonged to a college baseball team (baseball players, mean age: 21.5±1.4 years), and eight had no history of sporting activity (non-athletes, mean age: 21.8±1.8 years). All participants had good static visual acuity; i.e., equal to or better than 20/20. This study was approved by the ethical committee of the Faculty of Sports Sciences of Waseda University. Written informed consent was obtained from each participant after a detailed explanation of the experimental procedure and the object of the study.

### 2-2. Experimental design

**Figure 1:**
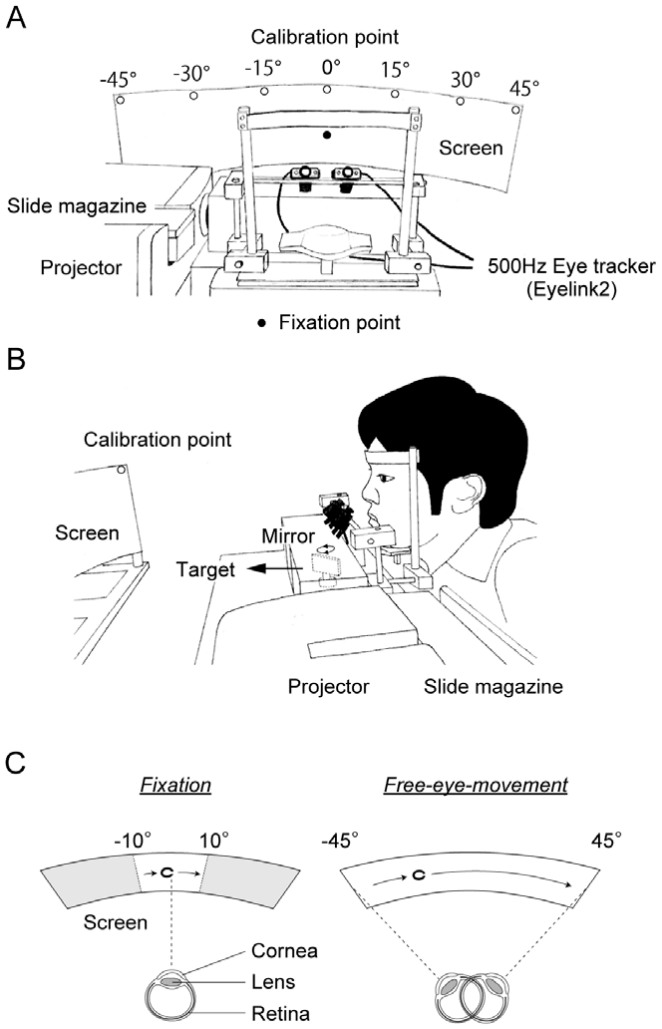
Experimental design. (A,B) Apparatus. (C) Two eye-movement conditions. Each participant placed their chin on a support, kept their head stationary, and followed a visual target that moved on a semi-circular screen (free-eye-movement conditions) or fixated on a fixation point in front them (fixation conditions).

The participants were seated facing a semicircular screen, which was placed 90 cm in front of them and occupied 90° of their visual field (Fig. 1A, B). A visual target (a Landolt “C” ring) was projected onto the screen by a slide projector. The motion of the target was controlled by rotating a mirror that was located between the projector and the screen (Fig. 1B). The participants were required to judge the direction of the gap (up, down, right, or left) in a forced choice manner, by pushing one of 4 buttons corresponding to each direction on a hand-held game controller.

In the first experiment, the target moved twice from one end to the other across the entire range of the screen at a constant speed, and the participants were allowed to move their eyes towards the target (free-eye-movement conditions). In the second experiment, a moving target was presented within a restricted range (±10° from the center of the screen) using an electromagnetic shutter, while the participants were required to fixate on a central fixation target (fixation conditions).

Eight different rings with two gap sizes (arc: 42 or 8 min) and 4 gap directions (up, down, right, or left) were used as targets. The target velocity was chosen from eight options (200, 300, 400, …, 900°/sec in the free-eye-movement conditions and 50, 100, 150, …, 400°/sec in the fixation conditions), and the movement direction was chosen from two options (left or right). Thus, there were 128 possible combinations (8 targets×8 speeds×2 directions) for each condition. Each combination was chosen once for each condition in a pseudorandom manner. The slide projector, shutter, and motor were automatically controlled using a digital IO board (PCI-7204; Interface Corp., JAPAN) and a homemade program (Visual C++; Microsoft Corp., USA) run on a Windows PC. After each trial, the participants responded by pushing a button on a handheld selector. Their responses were recorded by the PC.

In all participants, their eye movements during the task were measured at 500 Hz using an eye-tracker (Eyelink 2, SR Research Ltd.). Prior to each experiment, the system was calibrated by asking the participants to look at 7 calibration points, which were placed every 15 degrees along the screen (Fig. 1A).

### 2-3. Data analysis

The correct response rate was calculated for each group, for each target size and movement speed. The correct response rate (p) was fitted using the following psychometric function:
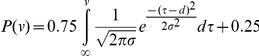
where v denotes the target speed, d represents the horizontal shift of the curve, and s is the width of the slope. MATLAB (optimization toolbox) was used for the fitting to minimize Pearson's chi-square statistic [22].

After fitting the curve to the data, the target speed at which a correct response rate of 75% was obtained was used as an objective measure of DVA; i.e., a higher speed reflects a better DVA.

## Results

### Typical example

**Figure 2:**
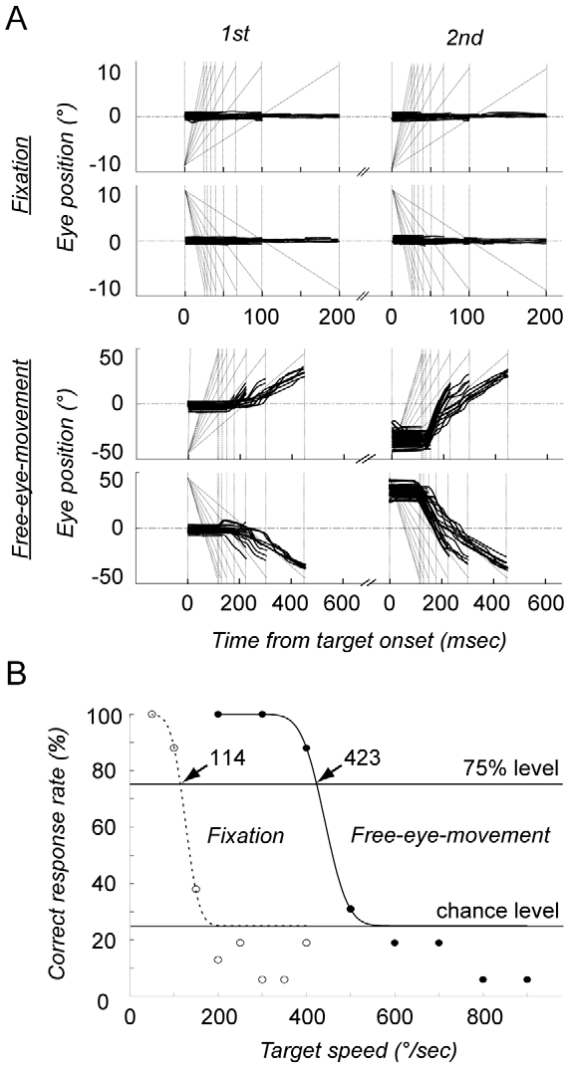
Data obtained from a typical participant (baseball player) using small targets. (A) Eye and target movements in the fixation (upper panels) and free-eye-movement conditions (lower panels). In each trial, the targets (oblique lines) traveled across the entire screen twice at a constant velocity. The participant's eye movements in the first and the second round are plotted separately in the left and the right columns, respectively. The participant did not move his eyes in the fixation conditions, but made catch-up saccades in the free-eye-movement conditions. (B) Correct response rates plotted against the target speed. The curves show the results of fitting to a psychometric function that saturates at 25% (chance level) and 100%. The intercepts of the curves at the 75% correct response rate are indicated by arrows.

**Figure 3:**
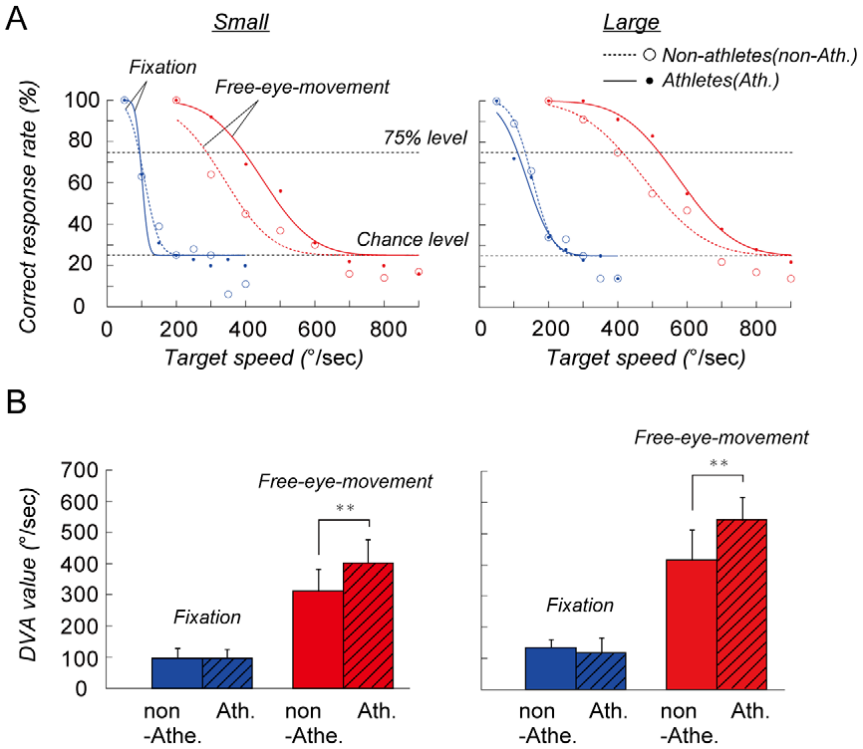
Group comparisons. (A) The mean correct response rate plotted against the target speed. The colors represent the fixation (blue) and free-eye-movement (red) conditions. The symbols the represent the non-athletes (open circles) and athletes (dots). Note the better performance of the athletes in the free-eye-movement conditions with both small (left) and large targets (right). (B) The mean target velocity that yielded a correct response rate of 75%. The threshold target velocity was used as a measure of DVA. Error bars show the standard deviation. **: p<0.01 (post hoc tests performed with the Ryan method).

Figure 2 shows the data for a typical participant (baseball player) for the small target. In the fixation conditions, his mean horizontal eye position was 0.02 degrees with a standard deviation of 0.84 degrees, indicating that he maintained almost complete fixation during the experiment (Fig. 2A, top panels). In the free-eye-movement conditions (Fig. 2A, bottom), the participant made a large predictive saccade to follow the target, especially in the second round (right columns). It is worth noting that in the second round the participant voluntarily waited for the target to appear at the edge of the screen (+45 or −45 degrees) and then made efficient catch-up saccades. Catch-up saccades were often followed by slower attempts of smooth pursuits, but the eyes were always left behind because the slowest target movement (200 deg/s) exceeded the maximum velocity of smooth pursuit eye movements (60 deg/s, [23]). The speed corresponding to a 75% correct response rate was 114°/sec in the fixation conditions and 423°/sec in the free-eye-movement conditions (Fig. 2B). This participant's DVA was approximately four times better in the free-eye-movement conditions than in the fixation conditions in terms of the threshold target velocity. When the data for each group of participants were pooled, it was found that the threshold speed in the free-eye-movement conditions was generally 3–5 times faster than that in the fixation conditions, (blue vs red traces in Fig. 3A).

### Group comparison

In the free-eye-movement conditions (red traces in Fig. 3A), the threshold speeds for the baseball players (396 and 520°/sec for the small and large target, respectively; solid curves) were higher than those of the non-athletes (286 and 413°/sec, respectively; dotted curves). However, in the fixation conditions (blue traces in Fig. 3A) the threshold speeds of the baseball players (94 and 110°/sec, for the small and the large target, respectively) were not very different from those of the non-athletes (92 and 133°/sec, respectively).

A two-way analysis of variance confirmed these observations. When two-way ANOVA (condition×group) was applied to the threshold speed for the small target (Fig. 3B, left), the main effect of condition (F_1,14_ = 225, p<0.0001) and the interaction of the two main effects (F_1,14_ = 6.41, p = 0.024) were significant. Post-hoc analyses showed that in the free-eye-movement conditions the mean threshold speed of the baseball players (404±74°/s, mean ± s.d.) was significantly faster than that of the non-athletes (315±69°/s, p = 0.001, Ryan's method [24]), but that the difference between their mean threshold speeds was not significant in the fixation conditions.

The results for the large target were basically the same (Fig. 3B, right). Two-way ANOVA showed that the main effect of condition (F_1,14_ = 253, p<0.0001) and the interaction of the two main effects (F_1,14_ = 10.4, p = 0.006) were significant. The post-hoc test showed that in the free-eye-movement conditions the mean threshold speed of the baseball players (545±74°/s) was significantly faster than that of the non-athletes (417±97°/s, p = 0.0003, Ryan's method).

### Relationship between the threshold speeds in the two conditions

**Figure 4:**
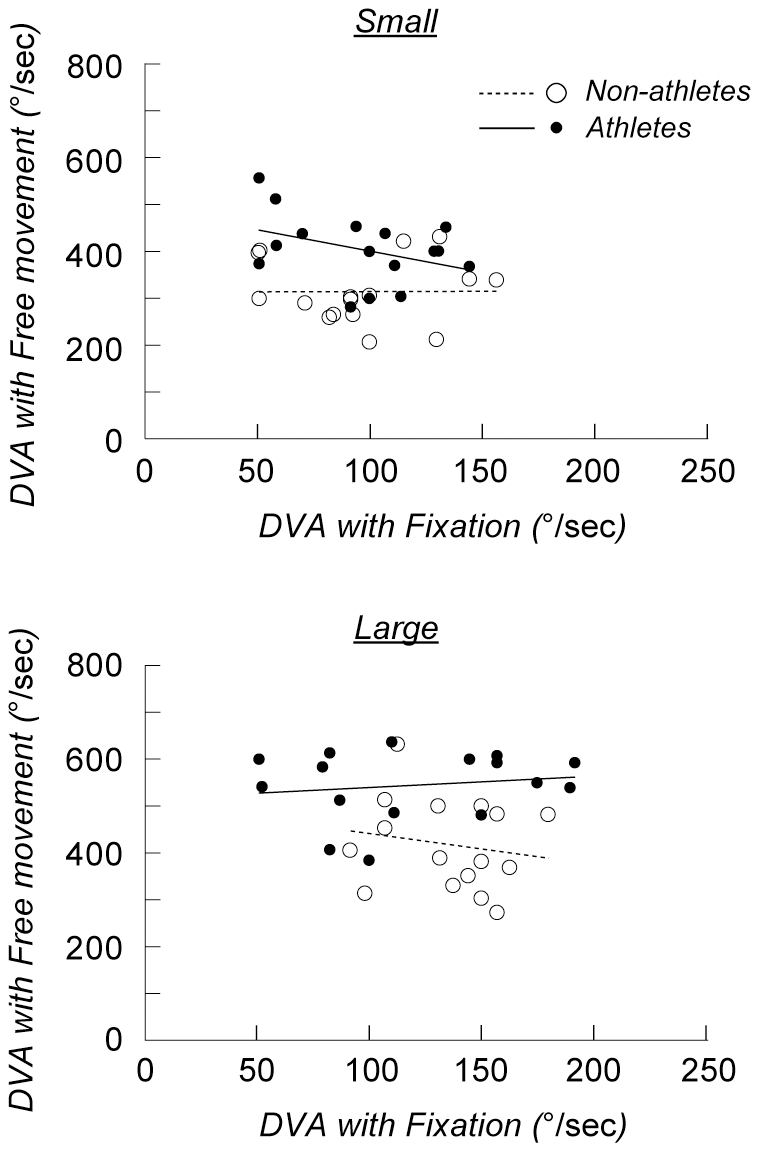
Correlations between DVA in the fixation and free-eye-movement conditions. DVA in the free-eye-movement conditions is plotted against DVA in the fixation conditions for each athlete (filled circles) and non-athlete (open circles). The lines show the results of the linear regression analysis. There was no significant correlation in either group for the small (top) or large targets (bottom) (p>0.1).

Although we did not find any difference between the groups in the fixation conditions, it was still possible that DVA in the free-eye-movement conditions was correlated with DVA in the fixation conditions within each group. However, there was no significant correlation in either group for either target size (Fig. 4).

## Discussion

To cancel out image blurring due to hand shaking, video cameras are equipped with anti-shaking mechanisms. A common anti-shaking approach is to prevent physical blurring on the sensor by moving the lens or the sensor itself. Another approach is to allow blurring on the sensor but then cancel it out by comparing successive images. In the present study, we hypothesized that humans employ both strategies to improve DVA and that athletes are better at both strategies; i.e., at tracking moving objects by moving their eyes and at perceiving moving objects from blurred images on their retinas. From our knowledge of perceptual learning of visual perception, we expected that athletes would be able to improve their perception of blurred images through perceptual learning of object movement during their daily training sessions. In this study, the DVA of the athletes was better than that of the non-athletes in the free-eye-movement conditions, but we did not find any difference between the groups in the fixation conditions. These results dismiss the second possibility that athletes are better at perceiving moving objects from blurred images on their retinas, and indicate that the better DVA of athletes is primarily due to an improved ability to track moving targets with their eyes.

However, the present results do not necessarily exclude the possibility that visual perceptual learning contributes to the visual performance of athletes in the games in which they specialize. Perceptual learning of visual perception generally occurs within the limited environment in which visual stimuli are presented [14], [25]. For example, perceptual learning during motion perception acquired through exposure to motion stimuli in one direction is not generally applicable to motion stimuli in others [15]–[17], [26]. Thus, if perceptual learning does have an effect on the DVA of athletes, it is most likely to be detected when moving objects are presented in a way that is similar to the way in which objects move in the game in which they specialize. For example, baseball players might show better DVA than non-athletes in fixation conditions when the direction and speed of the moving object are similar to those of a ball being thrown by a pitcher from the mound to the home base.

Another possible effect of perceptual learning is that better motion perception of the moving object when it was in the periphery of the screen led to better estimation of its future position and helped the athlete participants to make better anticipatory saccades to catch up with the moving object. These possibilities warrant more detailed investigation of perceptual abilities and eye movements.

## References

[1] Burg A, Hulbert S (1961). Dynamic visual acuity as related to age, sex, and static acuity.. Journal of Applied Psychology.

[2] Miller JW, Ludvigh EL (1962). The effect of relative motion on visual acuity.. Survey of Ophthalmology.

[3] Morris GSD, Kreighbaum E (1977). Dynamic visual acuity of varsity women volleyball and basketball players.. Research Quarterly.

[4] Rouse MW, DeLand P, Christian R, Hawley J (1988). A comparison study of dynamic visual acuity between athletes and nonathletes.. Journal of the American Optometric Association.

[5] Millslagle DG (2000). Dynamic visual acuity and coincidence-anticipation timing by experienced and inexperienced women players of fast pitch softball.. Perceptual and Motor Skills.

[6] Schneiders AG, Sullivan SJ, Rathbone EJ, Thayer AL, Wallis LM (2010). Visual acuity in young elite motorsport athletes: A preliminary report.. Physical Therapy in Sport.

[7] Sanderson FH, Whiting HTA (1974). Dynamic visual acuity and performance in a catching task.. Journal of Motor Behavior.

[8] Ishigaki H, Miyao M (1993). Differences in dynamic visual acuity between athletes and nonathletes.. Perceptual and Motor Skills.

[9] Bahill AT, Laritz T (1984). Why can't batters keep their eyes on the ball.. American Scientist.

[10] Land MF, McLeod P (2000). From eye movements to actions: how batsmen hit the ball.. Nature Neuroscience.

[11] Jacob R, Lillakas L, Irving EL (2005). Dynamics of saccadic adaptation: Differences between athletes and nonathletes.. Optometry and Vision Science.

[12] Bahill AT, Stark L (1979). The trajectories of saccadic eye movements.. Scientific American.

[13] Gibson JJ, Gibson EJ (1955). Perceptual learning; differentiation or enrichment?. Psychological Review.

[14] Gibson EJ (1963). Perceptual learning.. Annual review of psychology.

[15] Vaina LM, Belliveau JW, des Roziers EB, Zeffiro TA (1998). Neural systems underlying learning and representation of global motion.. Proceedings of the National Academy of Sciences of the United States of America.

[16] Watanabe T, Nanez JE, Koyama S, Mukai I, Liederman J (2002). Greater plasticity in lower-level than higher-level visual motion processing in a passive perceptual learning task.. Nature Neuroscience.

[17] Koyama S, Harner A, Watanabe T (2004). Task-dependent changes of the psychophysical motion-tuning functions in the course of perceptual learning.. Perception.

[18] Seitz AR, Nanez JE, Holloway SR, Watanabe T (2006). Perceptual learning of motion leads to faster flicker perception.. Plos One.

[19] Watanabe T, Nanez JE, Sasaki Y (2001). Perceptual learning without perception.. Nature.

[20] Westheimer G, Mckee SP (1975). Visual acuity in presence of retinal-image motion.. Journal of the Optical Society of America.

[21] Demer JL, Amjadi F (1993). Dynamic visual acuity of normal subjects during vertical optotype and head motion.. Investigative Ophthalmology & Visual Science.

[22] Linhart H, Zucchini W (1986). Model Selection.

[23] Schalen L (1980). Quantification of tracking eye movements in normal subjects.. Acta Otolaryngologica.

[24] Day RW, Quinn GP (1989). Comparisons of treatments after an analysis of variance in ecology.. Ecological Monographs.

[25] Schoups A, Vogels R, Qian N, Orban G (2001). Practising orientation identification improves orientation coding in V1 neurons.. Nature.

[26] Ball K, Sekuler R (1981). Adaptive processing of visual motion.. Journal of Experimental Psychology-Human Perception and Performance.

